# Fatty Acid Metabolism via CPT1A Supports Poll Gland Function and Rutting Activities in Male Bactrian Camels

**DOI:** 10.3390/biom15070988

**Published:** 2025-07-11

**Authors:** Qi Ma, Bohao Zhang, Bin Zhou, Quanwei Zhang, Yuan Gao

**Affiliations:** 1 College of Life Science and Technology, Gansu Agriculture University, Lanzhou 730070, China; maq@gsau.edu.cn (Q.M.); zhoub@st.gsau.edu.cn (B.Z.); 2 College of Veterinary Medicine, Gansu Agriculture University, Lanzhou 730070, China; zhangbh@st.gsau.edu.cn; 3 Gansu Key Laboratory of Animal Generational Physiology and Reproductive Regulation, Lanzhou 730070, China

**Keywords:** Bactrian camel, poll gland, CPT1A, fatty acid, breeding, energy metabolism

## Abstract

The poll gland, a specialized tissue of male Bactrian camels, undergoes seasonal enlargement and marked metabolic activation during the rutting season. However, the metabolic mechanisms of the poll gland and its role in rutting activities and inducing estrus are still not fully understood. This study aimed to investigate the contribution of fatty acid metabolic pathways, specifically those mediated by carnitine palmitoyltransferase 1A (CPT1A), in poll gland activity during the breeding season; poll gland tissue, neck mane, and urine samples were systematically collected from healthy male Bactrian camels stratified into breeding and non-breeding season groups for integrated proteomic, metabolomic, and biochemical assays. Histological and immunohistochemical analyses revealed reduced adipocytes but elevated ATP production in rutting camels, suggesting increased mitochondrial activity and enhanced oxidative phosphorylation. Proteomic analyses identified 119 differentially expressed proteins (DEPs) linked to fatty acid metabolism, with CPT1A, a key regulator of mitochondrial fatty acid oxidation, emerging as a central hub. The Kyoto Encyclopedia of Genes and Genomes (KEGG) pathway analysis further confirmed enrichment in fatty acid biosynthesis, degradation, and PPAR/AMPK signaling. The metabolomic analysis identified 14 metabolites, including acetylcarnitine and glycine, that were closely correlated with CPT1A expression, suggesting their potential involvement in regulating fatty acid metabolism during the breeding season. Quantitative expression analyses revealed that CPT1A in glandular acini was significantly upregulated in the breeding group compared to the non-breeding group across all assays: qPCR (2.53-fold, *p* < 0.05), Western blot (3.5-fold, *p* < 0.05), and immunohistochemistry (1.5-fold, *p* < 0.05). This demonstrated that CPT1A-mediated fatty acid metabolism plays a pivotal role in energy provision for reproductive activities. The results suggested that CPT1A-mediated fatty acid oxidation sustains poll gland function and reproductive behaviors in male Bactrian camels. This study provided a theoretical basis for understanding the role of CPT1A-mediated fatty acid oxidation in maintaining poll gland function and supporting reproductive activities in male Bactrian camels.

## 1. Introduction

The Bactrian camel (*Camelus bactrianus*), a critically important livestock species in arid and semi-arid ecosystems, is facing a global decline in reproductive efficiency. Although recent investigations employing Bactrian camels have identified several biomarkers associated with reproductive and metabolic processes [1,2], the comprehensive physiological and molecular mechanisms regulating camel reproduction remain incompletely characterized. The camel’s reproductive physiology represents a complex adaptive system shaped by desert selection pressures [3], integrating multiple specialized features: seasonal breeding cyclicity, induced ovulation mechanisms, one of the longest gestation periods among terrestrial mammals, and evolutionarily constrained reproductive rates [3,4,5]. These specialized adaptations, while optimizing individual fitness under xeric conditions with limited resources, create fundamental constraints on reproductive output that increasingly jeopardize population sustainability amid growing climatic challenges and human-induced pressures [6,7]. Male camels have a characteristic poll gland (occipital gland or neck occipital gland), a V-shaped structure located bilaterally at the C1 vertebra within the dermis [8]. Composed of pyramidal lobules with almond-like pigmentation, this gland undergoes seasonal enlargement during rutting seasons, secreting a viscous, amber-colored fluid with a potent odor [8,9]. The poll gland’s secretions, rich in bioactive compounds, play pivotal roles in estrus induction and mating facilitation by stimulating female camels and enhancing male sexual activity [10].

During rutting, males exhibit distinct behavioral displays, including tail-urine flapping and defecation, vocalizations (“beeping”), mucus secretion, and territorial aggression [6,11]. These energetically demanding activities correlate with increased gland development and pheromone output, suggesting a direct link between poll gland function and reproductive success [10,12]. Additionally, behaviors such as foaming at the mouth, teeth grinding, and reduced appetite during rutting may reflect metabolic trade-offs between reproductive effort and energy conservation. Although the poll gland’s functional significance is well-established, the underlying molecular mechanisms coordinating its activity with pheromone production, metabolic processes, and mating behaviors are yet to be fully elucidated. Addressing these knowledge gaps may contribute to the development of strategies aimed at improving camel fertility and supporting the species’ ecological and economic roles. Fatty acids provide a more sustained energy source than carbohydrates and proteins, making fatty acid metabolism especially important during energy-demanding physiological states such as the rutting season in male Bactrian camels, when prolonged physical activity and poll gland secretory function are pronounced [13]. This study focuses on fatty acid catabolism, particularly the mitochondrial β-oxidation pathway, to investigate how metabolic energy supports reproductive behaviors.

Fatty acid catabolism, a central energy-producing pathway, involves sequential enzymatic steps: lipolysis, mitochondrial transport via carnitine palmitoyltransferase 1A (CPT1A), β-oxidatiolhyufon, and acetyl-CoA generation for ATP synthesis [14,15]. CPT1A, a rate-limiting enzyme, facilitates fatty acid entry into mitochondria, while acyl-CoA synthetases (ACSLs) activate long-chain fatty acids [16]. Hydroxyacyl-CoA dehydrogenase (HADH) and other β-oxidation enzymes contribute to acetyl-CoA production, linking lipid catabolism to the tricarboxylic acid (TCA) cycle [17,18]. CPT1A serves as a master regulator of this pathway, ensuring metabolic flexibility and energy homeostasis, particularly during fasting or high energy demand [19]. CPT1A activity is tightly controlled by nutritional status and hormonal cues such as glucagon insulin and malonyl-CoA, allowing cells to adapt fuel usage to metabolic needs [20]. In brown adipose tissue, CPT1A-mediated fatty acid oxidation contributes to thermogenesis through uncoupling protein 1 (UCP1), while in the liver, it supports ketogenesis and helps maintain systemic glucose balance during energy deprivation [2,3]. In brown adipose tissue, CPT1A-mediated fatty acid oxidation contributes to thermogenesis through uncoupling protein 1 (UCP1), while in the liver, it supports ketogenesis and helps maintain systemic glucose balance during energy deprivation [2,3]. These functions highlight the cross-tissue relevance of CPT1A in maintaining energy homeostasis. Moreover, dysregulation of CPT1A has been implicated in various metabolic disorders, including hepatic steatosis and insulin resistance, underscoring its clinical significance. Pharmacological activation of CPT1A, for example via AMPK/mTORC1 pathway modulators, has shown therapeutic promise in improving lipid metabolism and alleviating metabolic syndromes [4,21,22].

This study aims to investigate the molecular mechanisms of fatty acid metabolism, particularly the role of CPT1A, in the poll gland of male Bactrian camels during the rutting season. Specifically, proteomic and metabolomic analyses were employed to identify differentially expressed proteins (DEPs) and metabolites associated with reproductive behaviors. The expression and localization changes of CPT1A in the glandular tissues were evaluated to understand its role in metabolic fueling and reproductive activities. Our findings provide new insights into the patterns of fatty acid metabolism in the poll gland and reproduction of male Bactrian camels.

## 2. Materials and Methods

### 2.1. Animals and Samples

For this study, healthy male Bactrian camels (*Camelus bactrianus*) of similar age (8 years old) and body weight (480 ± 5 kg) were selected from a farm in Zhangye (Gansu, China). During the non-breeding season (April to November), the camels were allowed to roam and feed freely, while they were kept in captivity during the breeding season (December to March of the following year). To minimize animal suffering, candidate camels were deeply sedated by intravenous injection of Xylazine (0.3 mg/kg, Lanzhou, China) and then induced to painless death via intravenous injection of pentobarbital sodium (140 mg/kg, Dechra Veterinary Products, Shrewsbury, UK). Samples of neck mane and urine were collected in June (non-breeding season, *n* = 3) and January (breeding season, *n* = 3), serving as the non-breeding season group (NBS group) and breeding season group (BS group), respectively. Due to significant atrophy of the poll gland during the non-breeding season, tissue collection was only feasible during the breeding season. Poll gland tissues with high and low secretion levels were collected accordingly. All samples were processed as follows: flash-frozen in liquid nitrogen for proteomic analysis and fixed in 4% paraformaldehyde for histochemical examination. Then, neck mane, urine, and poll gland samples from different reproductive stages were used for a non-targeted metabolomic analysis. All procedures strictly adhered to the ethical guidelines approved by the Animal Protection Committee of Gansu Agricultural University (Approval No. GSU-LC-2020-39).

### 2.2. Metabolite Extraction and Liquid Chromatography–Mass Spectrometry (LC-MS) Analysis

Poll gland tissue or neck mane samples (80 mg) were homogenized in 200 μL of ultrapure water (H_2_O) using a homogenizer (Wonbio, Shanghai, China) equipped with five ceramic beads. For metabolite extraction, 800 μL of a methanol–acetonitrile (1:1, *v*/*v*) solution was added to the homogenate or urine samples. The mixture was vortexed thoroughly and then centrifuged at 12,000× *g* for 15 min. The resulting supernatant was collected and dried under vacuum using a centrifuge (Bioridge, Shanghai, China). The dried residue was reconstituted in 100 μL of acetonitrile–water (1:1, *v*/*v*) for subsequent LC-MS analysis. Metabolite profiling was performed using ultra-high-performance liquid chromatography (UHPLC; 1290 Infinity LC, Agilent Technologies, Santa Clara, CA, USA) coupled with a high-resolution mass spectrometer (AB Sciex TripleTOF 6600, Waters, Redwood City, CA, USA). Chromatographic separation was achieved on an ACQUITY UPLC HSS T3 column (100 mm × 2.1 mm, 1.8 μm; Waters, Redwood City, CA, USA). To ensure analytical reproducibility, quality control (QC) samples were prepared by pooling equal volumes of all extracted samples and analyzed intermittently throughout the run. Raw LC-MS data were processed using Progenesis QI software v.4.2 (Nonlinear Dynamics, Newcastle, Newcastle upon Tyne, UK) for baseline correction, peak alignment, deconvolution, and peak area integration. Metabolite identification was performed by matching mass spectra against the Human Metabolome Database (HMDB). Differentially expressed metabolites (DEMs) were screened based on a false discovery rate (FDR) < 0.05 and a variable importance in projection (VIP) score > 1. Identified metabolites were functionally annotated and subjected to pathway enrichment analysis using the Kyoto Encyclopedia of Genes and Genomes (KEGG) database.

### 2.3. Proteomic Sequencing and Bioinformatics Analysis

Tissue samples were processed according to the manufacturer’s protocol for the iST Sample Preparation Kit (PreOmics, Tübingen, Germany). The workflow included protein denaturation, reduction with dithiothreitol (DTT), alkylation with iodoacetamide (IAA), tryptic digestion, and peptide cleanup using C18 spin columns. For spectral library generation, digested peptides were reconstituted in buffer (20 mM ammonium formate in water, pH 10.0) and fractionated by high-pH reversed-phase chromatography on an Ultimate 3000 system (ThermoFisher, Waltham, MA, USA) equipped with an XBridge C18 column (3.5 μm, 4.6 × 150 mm; Waters, Waltham, MA, USA). Peptides were eluted using a 5–45% gradient of buffer (20 mM ammonium formate in 80% acetonitrile, pH 10.0) over 60 min, with collection of six fractions. Fractionated peptides were analyzed by LC-MS using an Orbitrap Lumos mass spectrometer (ThermoFisher, Waltham, MA, USA) in data-independent acquisition (DIA) mode. Raw DIA files were processed using Spectronaut X (Biognosys AG, Schlieren, Switzerland) with default settings. DEPs were identified using thresholds of a false discovery rate (FDR) < 0.05 and a log2 fold change > 0.58. The complete DIA proteomic dataset was deposited into the ProteomeXchange Consortium via the PRIDE repository with the dataset identifier PXD047457.

To characterize the biological functions of DEPs, Gene Ontology (GO) annotation and KEGG enrichment analyses were performed with particular focus on proteins associated with fatty acid catabolism. For data visualization, hierarchical clustering heatmaps and Venn diagrams were generated using the OmicShare network (https://www.omicshare.com/tools/). Protein–protein interaction networks were constructed by integrating data from the STRING database (version 11.5, https://cn.string-db.org/) and visualized using Cytoscape software (version 3.9.1).

### 2.4. RNA Isolation and Quantitative Gene Expression Analysis

Total RNA was isolated from tissue samples using the TRIzol reagent (Invitrogen) according to the manufacturer’s protocol. RNA concentration and purity were determined spectrophotometrically (NanoDrop 2000, ThermoFisher, Waltham, MA, USA). First-strand cDNA was synthesized from 1 μg total RNA using a PrimeScript RT reagent kit (Takara, Dalian, China) following the manufacturer’s instructions. Quantitative real-time PCR (qPCR) was performed using SYBR Premix Ex Taq (Takara) on a QuantStudio 6 Flex Real-Time PCR System (Applied Biosystems). The thermal cycling conditions consisted of 95 °C for 30 s followed by 40 cycles of 95 °C for 5 s and 60 °C for 30 s. A melt curve analysis was performed to verify amplification specificity. The qPCR primer sequences used in this study were as follows: CPT1A F, 5′-ATTTCCTCCCGGTCCAGTTT-3′; CPT1A R, 5′-GGACAGCAAGCACATAGTCG-3′; β-actin F, 5′-CCAAGGCCAACCGTGAGAA-3′; β-actin R, 5′-CCAGAGGCATACAGGGACAG-3′. All primers were custom-synthesized by Tsingke Biotechnology (Yangling, China). *β-actin* served as the endogenous control for normalization. Relative gene expression levels were calculated using the 2^−ΔΔCT^ method. All experiments were performed in triplicate with three biological replicates.

### 2.5. Histochemical Analysis

Paraffin-embedded fixed tissues were sectioned at 5 µm thickness using a rotary microtome (Leica RM2235, Wetzlar, Germany). Tissue sections were subjected to standard hematoxylin and eosin (H&E) staining and immunohistochemical (IHC) analysis following established protocols [2,12]. For IHC staining, sections were incubated with the following primary antibodies: rabbit anti-COX IV (1:500 dilution; Proteintech, Wuhan, China) and rabbit anti-CPT1A (1:400 dilution; Proteintech). Stained sections were digitally imaged using a high-resolution whole-slide scanner (Pannoramic, 3D HISTECH, Budapest, Hungary). All experiments were performed in triplicate to ensure reproducibility.

### 2.6. Immunofluorescence (IF) Analysis

IF staining was performed following established protocols [12]. Tissue sections were incubated with the following primary antibodies: rabbit anti-COX IV (1:300 dilution; Proteintech), rabbit anti-CPT1A (1:300 dilution; Proteintech), and mouse monoclonal anti-CK7 (1:500; Bioss, Beijing, China). After secondary antibody incubation, cell nuclei were counterstained with 4′,6-diamidino-2-phenylindole (DAPI; 1 μg/mL, Solarbio, Beijing, China) for 10 min at room temperature. Fluorescence images were acquired using a high-resolution fluorescence microscope (Echo Revolve R4, Echo Laboratories, San Diego, CA, USA) equipped with appropriate filter sets. All experiments were repeated independently at least three times.

### 2.7. Western Blot Analysis

Protein expression levels were analyzed by Western blotting following established protocols. In brief, tissue samples were homogenized in RIPA lysis buffer containing protease inhibitors (Beyotime, Beijing, China), and protein concentrations were determined using the BCA assay (Beyotime). Equal amounts of protein (30 μg per lane) were separated by 10% SDS-PAGE and transferred to PVDF membranes (Millipore, Billerica, MA, USA). Membranes were blocked with 5% skimmed milk in TBST for 1 h at room temperature then incubated overnight at 4 °C with the following primary antibodies: rabbit polyclonal anti-CPT1A (1:5000; Proteintech) and mouse monoclonal anti-β-actin (1:8000; Bioss). After incubation with HRP-conjugated secondary antibodies (1:3000; Proteintech) for 1 h at room temperature, protein bands were visualized using an enhanced chemiluminescence (ECL) substrate (Beyotime). Band intensities were quantified using ImageJ software v1.44p (NIH; Bethesda, MD, USA). β-actin served as the loading control for normalization. All experiments were repeated three times with independent biological replicates.

### 2.8. Quantitative Biochemical Analyses

ATP concentration in tissues was quantified using an ATP content detection kit (chemiluminescence method, G4309, Servicebio, Wuhan, China) following the manufacturer’s instructions. Catalase (CAT) activity in tissues was assessed with a CAT detection kit (G4307, Servicebio) in accordance with the manufacture’s protocols.

### 2.9. Statistical Analysis

All statistical analyses were conducted using SPSS statistical software (version 26.0, IBM Corporation, Chicago, IL, USA). Continuous variables were expressed as mean ± standard deviation (SD) and analyzed using two-tailed Student’s t-tests for comparisons between groups. For multiple comparisons, one-way ANOVA followed by Tukey’s post hoc test was applied. Data visualization was performed using OriginPro software (version 9.1, OriginLab Corporation, Northampton, MA, USA). A probability value (*p*) of less than 0.05 was considered statistically significant for all analyses.

## 3. Results

### 3.1. Breeding Season Induces Significant Energy Production Increase in Camel Poll Gland

**Figure 1:**
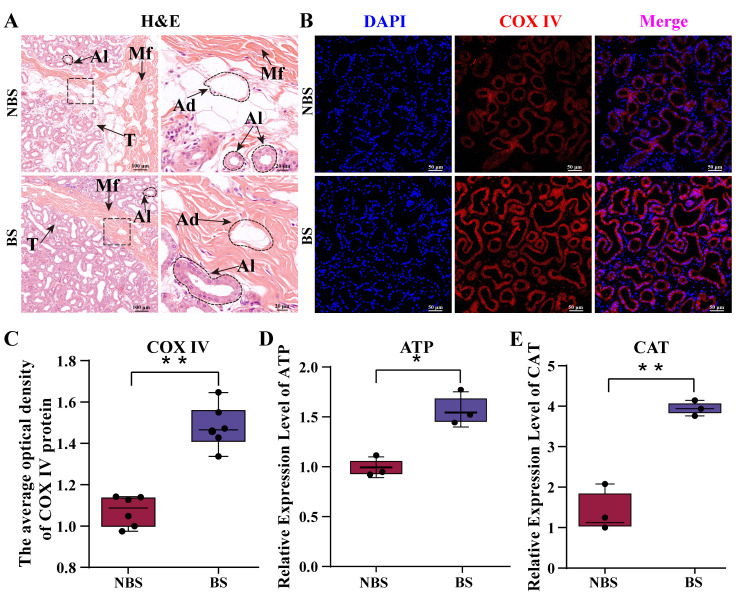
Breeding season induces significant energy production increase in camel poll gland. (**A**) Hematoxylin and eosin (H&E) staining of camel poll gland tissue during non-breeding and breeding season. The right column image is an enlarged view of the box area in the left column image. (**B**) Immunohistochemical (IHC) staining of cytochrome c oxidase subunit IV (COX IV) in camel poll gland tissue during non-breeding and breeding seasons. (**C**) The relative optical density of COX IV fluorescence in B. (**D**) ATP content in poll gland tissue was detected using a chemiluminescence assay kit. (**E**) Catalase (CAT) activity in poll gland tissue was measured by a CAT detection kit. NBS: poll glands during non-breeding season. BS: poll glands group during breeding season. Ad: adipocyte. AI: acinar. Mf: muscle fibers. Data are presented as means ± SEM; * represents *p <* 0.05 and ** represents *p* < 0.01.

H&E staining revealed distinct histological features and adipocyte distribution patterns in the camel poll gland across reproductive stages. The IHC analysis was performed using antibodies against cytochrome c oxidase subunit IV (COX IV), a well-established marker for cellular energy metabolism levels [23]. ATP concentration and CAT activity were measured by quantitative biochemical analyses, respectively. As shown in Figure 1A, the histological morphology of the poll gland in the NBS group revealed abundant thin interstitial connective tissue that separates the acini, with numerous adipocytes presented in the peri-acinar regions. These adipocytes exhibited well-defined borders, rounded morphology, and large, intact lipid droplets that were distributed among muscle fibers (Figure 1A). In contrast, during the non-breeding season, the histological analysis showed a reduction in adipocyte presence around the acini. The adipocytes that were still present had a shrunken cell shape, smaller lipid droplets, and a less distinct morphology, indicating a significant decrease in fat stores during this period (Figure 1A). These observations suggest a clear seasonal variation in the composition and structure of the poll gland tissue, linked to energy storage and expenditure during the breeding cycle. The IF staining showed that the COX IV fluorescence signal intensity in the BS group was stronger than that in the NBS group, about 1.5 times higher (Figure 1B,C). Moreover, the quantitative biochemical analyses of ATP and CAT in the poll gland tissue samples showed that the ATP concentration and CAT activity increased significantly in the BS group (Figure 1D,E). The results suggested that the adipose cells in the dromedary camel poll gland decrease, while substantial ATP is produced during the breeding season, which may be associated with the regulation of energy metabolism during the breeding season.

### 3.2. Identification of Candidate DEPs Related to Fatty Acid Metabolism by Using GO Annotation

**Figure 2:**
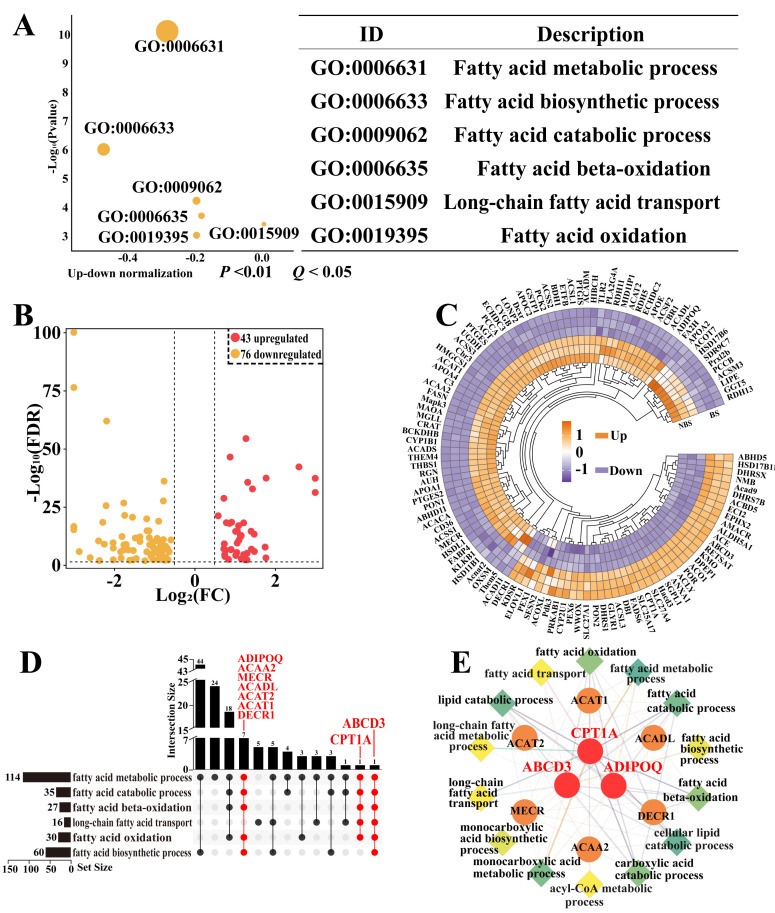
Identification of biological processes (BPs) associated with fatty acid metabolism and enrichment analysis. (**A**) Enrichment analysis (*p* < 0.05 and adjusted *p* < 0.05) identified six biological processes (BPs) associated with fatty acid metabolism. (**B**) Volcano plot analysis of differentially expressed genes across the six enriched BPs in (**A**). (**C**) Heatmap clustering of differentially expressed genes across the six enriched BPs in (**A**). (**D**) Clue GO analysis of nine key proteins involved in all six enriched BPs. (**E**) Protein–protein interaction (PPI) network construction of nine key proteins and six enriched BPs.

The DIA proteomic analysis identified differentially expressed proteins (DEPs) related to fatty acid metabolism in the poll glands of male Bactrian camels. The GO functional annotation revealed the significant enrichment of six biological processes (BPs) related to fatty acid metabolism (*p* < 0.05 and adjusted *p* < 0.05), including the fatty acid metabolic process, the biosynthetic process, the catabolic process, transport, and oxidation (Table S1, Figure 2A), indicating that breeding activities significantly promote fatty acid metabolism in the poll gland. A total of 119 DEPs were identified across these six BPs (Table S2), with 43 DEPs showing upregulation and 76 DEPs exhibiting downregulation (Figure 2B,C). The gene identification analysis revealed that nine key genes, ABCD3, CPT1A, ADIPOQ, ACAA2, MECR, ACADL, ACAT2, ACAT1, and DECR1, were consistently involved in all six enriched biological processes. Notably, CPT1A, ABCD3, and ADIPOQ demonstrated the most significant enrichment in fatty acid metabolic pathways and were identified as core DEPs, exhibiting extensive protein–protein interactions with other candidate DEPs (Figure 2D,E).

### 3.3. Identification of Candidate DEPs Related to Fatty Acid Metabolism from KEGG Pathway

**Figure 3:**
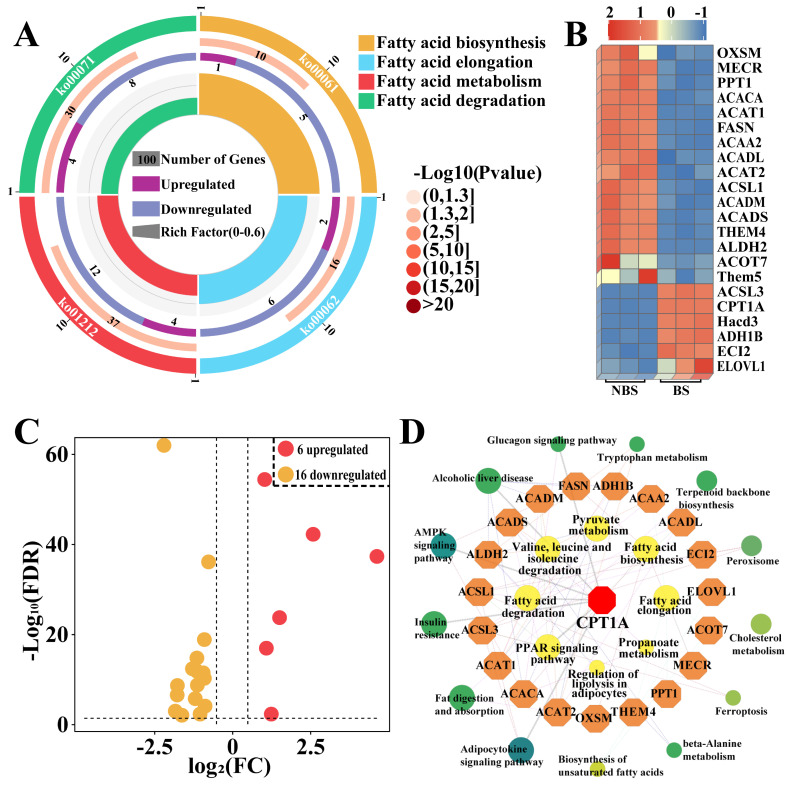
Identification of pathways associated with fatty acid metabolism and enrichment analysis. (**A**) Enrichment analysis identified four differential pathways associated with fatty acid metabolism. (**B**) Heatmap clustering of DEPs across the four pathways in (**A**). (**C**) Volcano plot analysis of DEPs across the four pathways in (**A**). (**D**) Protein–protein interaction (PPI) network construction of 22 DEPs and four pathways.

To further identity the differential signaling pathways in the poll glands of male Bactrian camels during the breeding season, this study performed a KEGG pathway analysis to screen differential pathways related to fatty acid metabolism based on DIA proteomic data. As shown in Figure 3, the enrichment analysis (*p* < 0.05 and adjusted *p* < 0.05) identified four differential pathways associated with fatty acid metabolism (Table S3), including fatty acid biosynthesis, elongation, metabolism, and degradation (Figure 3A), which is consistent with the GO enrichment analysis. There are a total of 22 DEPs in the four pathways (Table S4), of which 6 DEPs are upregulated and 16 DEPs are downregulated (Figure 3B,C). The correlation analysis of 22 DEPs indicated that CPT1A is the most involved in fatty acid-related biological processes and is the core gene interacting with other candidate DEPs (Figure 3D).

### 3.4. Integrated Screening of Key Regulators in Fatty Acid Metabolism

**Figure 4:**
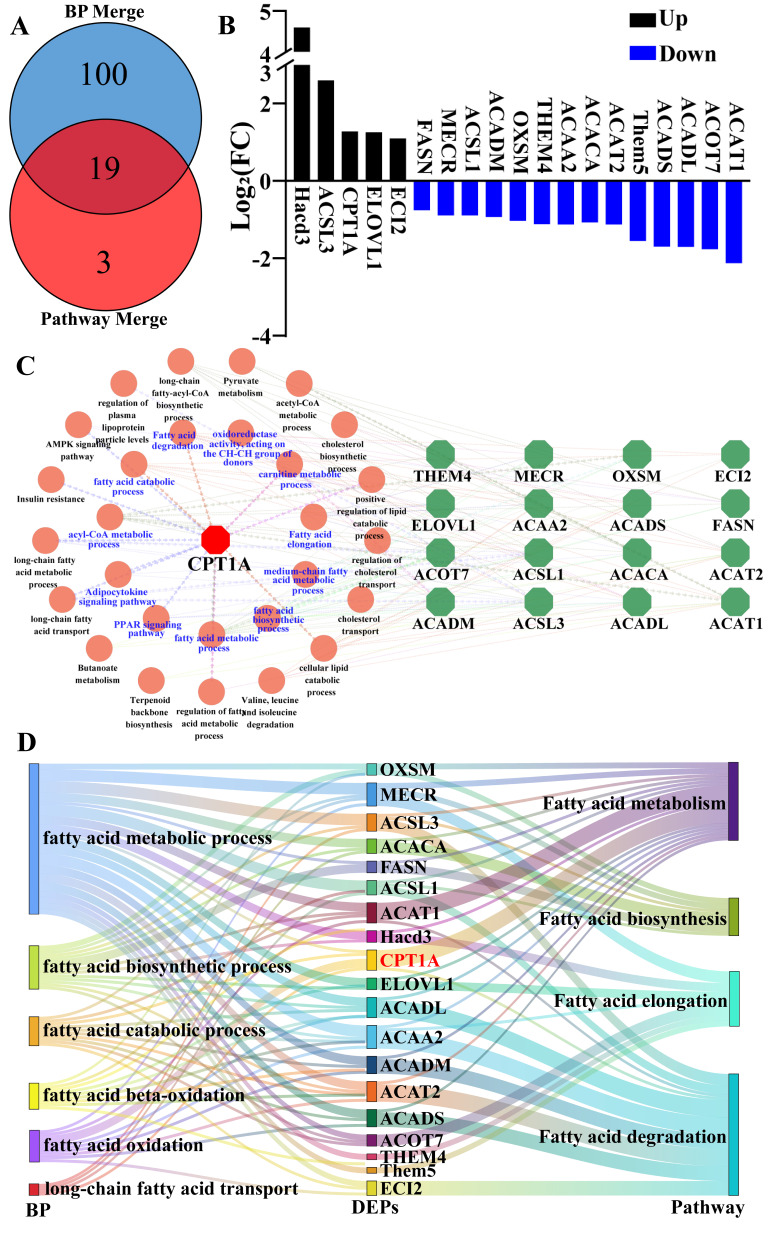
Integrated screening of key regulators in fatty acid metabolism. (**A**) Venn diagram identified key regulatory proteins from 119 DEPs of GO terms and 22 DEPs of pathways involved in fatty acid metabolism. (**B**) Expression changes of 19 proteins in DIA proteomic analysis. (**C**) Protein–protein interaction (PPI) network construction of 19 DEPs and fatty acid metabolism-related pathways. (**D**) The Sankey diagram constructs the relationship between DEPs, four pathways, and six biological processes related to fatty acid metabolism.

To identify potential key regulators of fatty acid metabolism, this study performed an integrated analysis of 119 DEPs associated with GO terms and 22 DEPs linked to pathways. As shown in Figure 4, a subset of 19 DEPs overlapped between the GO enrichment and pathway analysis (Figure 4A), including 5 upregulated and 14 downregulated proteins (Figure 4B). The PPI construction of these 19 DEPs showed that CPT1A is involved in multiple biological processes of fatty acid metabolism and is a core factor interacting with candidate DEPs (Figure 4C,D).

### 3.5. Correlation Analysis Between CPT1A and Differential Metabolites in Tissue Samples

**Figure 5:**
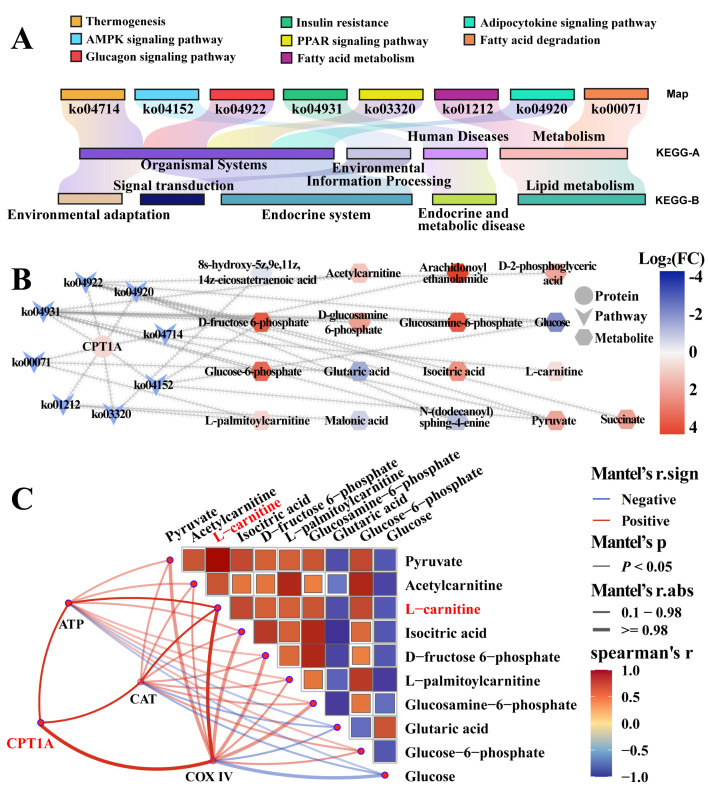
Correlation analysis between CPT1A and DEMs in tissue samples. (**A**) Screen of signaling pathways closely related to CPT1A from metabolite profiling data. (**B**) Interaction network construction of CPT1A, differential signaling pathways, and DEMs. (**C**) Correlation analysis between CPT1A and DEMs in tissue samples.

Previous research has identified CPT1A as a key regulatory factor in fatty acid metabolism. To further explore the role of CPT1A in influencing metabolite content during the breeding season, this study performed a correlation analysis between CPT1A and differential metabolites in tissue samples. As shown in Figure 5, there are eight significantly regulated signaling pathways closely related to CPT1A (Table S5, namely thermogenesis (ko04714): the PPAR signaling pathway (ko03320), the AMPK signaling pathway (ko04152), fatty acid metabolism (ko01212), the glucagon signaling pathway (ko04922), the adipocytokine signaling pathway (ko04920), insulin resistance (ko04931), and fatty acid degradation (ko00071) (Figure 5A). Among them, 14 DEMs are involved, with 9 metabolites showing upregulation and 5 metabolites exhibiting downregulation (Figure 5B). Further analysis of the correlation between CPT1A and DEMs showed that CPT1 expression was significantly positively correlated with acetylcarnitine and glycine levels (Figure 5C). These results provide a foundation for subsequent experimental verification.

### 3.6. Expression and Distribution of CPT1A in Poll Gland During Breeding Season

**Figure 6:**
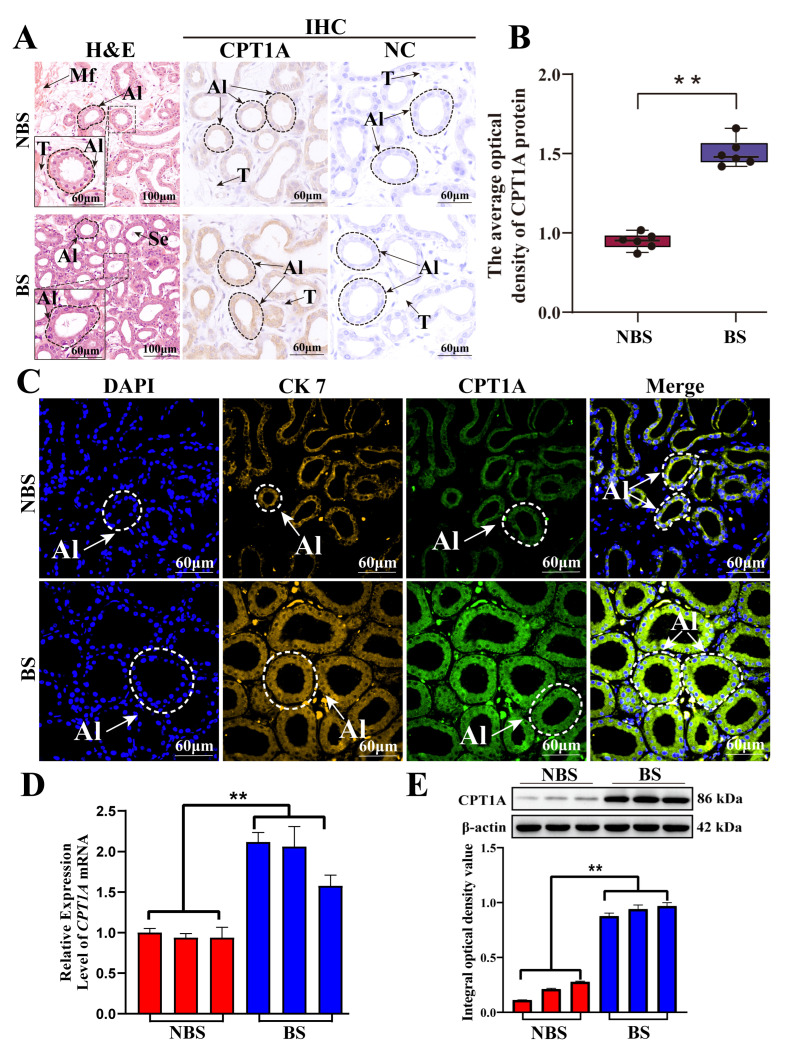
Expression and distribution of CPT1A in poll gland tissue. (**A**) Histological examination of poll gland tissue was performed using H&E staining, while CPT1A localization was assessed via immunohistochemical (IHC) analysis. (**B**) The optical density of CPT1A was quantified from IHC staining. (**C**) The immunofluorescence (IF) staining of poll glands using anti-CPT1A and CK7 antibodies, respectively. Cell nucleus stained blue with DAPI. (**D**) The mRNA levels of *CPT1A* were monitored by qPCR assay. (**E**) The protein expression levels of *CPT1A* were monitored by Western blot assay, and the optical density of bands. AI: acinar; Se: secretions; Mf: muscle fibers. ** *p* < 0.01. Original images of western blot can be found in Figures S1 and S2.

To further validate the CPT1A expression and distribution in male Bactrian camel poll gland tissues during the breeding season, this study assessed its protein localization and gene expression levels using qPCR, Western blot, IHC, and IF assays. As shown in Figure 6, CPT1A is mainly expressed in acinar epithelial cells by IHC staining (Figure 6A). And CPT1A showed co-localization with the epithelial cell marker CK7 in acinar (Figure 6C). The intensity of CPT1A immunostaining is significantly higher in the BS group compared to the NBS group (Figure 6A–C), with a 1.5-fold increase observed by quantitative IHC analysis (*p* < 0.05), suggesting that the expression of CPT1A significantly increased during breeding season. Consistently, qPCR and Western blot analyses demonstrated concordant upregulation of CPT1A expression at both transcriptional and translational levels, showing 2.53-fold and 3.5-fold increases, respectively, in the BS group relative to the NBS group (*p* < 0.05) (Figure 6D,E). Collectively, these results demonstrated that CPT1A is widely expressed in poll gland tissue and significantly activated during the breeding season, which may play an important regulatory role in the breeding activity of male Bactrian camels.

## 4. Discussion

As seasonal breeders, Bactrian camels exhibit distinct seasonal breeding patterns, with reproductive activity primarily occurring during the winter months. This reproductive seasonality is particularly evident in male camels, where the development and secretory activity of poll glands show marked cyclical changes synchronized with sexual activity [4,10,24]. The poll gland, a specialized sebaceous structure located in the cranial region, produces pheromone-rich secretions that serve as crucial chemical signals in camel reproduction [8,9]. These secretions contain bioactive compounds that not only enhance male libido but also play a key role in stimulating estrus behavior and mating receptivity in female camels [25]. However, the precise biochemical composition and mechanistic functions of poll gland secretions remain poorly understood. The specific pheromonal compounds responsible for mediating reproductive behaviors and their mode of action at the molecular level have yet to be fully characterized.

Seasonal breeding in male animals is typically associated with increased energy demands due to courtship displays, mate competition, and reproductive behaviors [26]. This general trend is also evident in male Bactrian camels, whose poll gland undergoes marked seasonal activation. Our group is committed to exploring the metabolites of the camel poll gland and their roles in regulating the breeding season via identifying DEMs and DEPS. Our previous study identified some differential metabolites and clarified the metabolic mechanism in the poll gland, such as hydrogen sulfide (H_2_S) and creatine [1,10,12]. In this study, we found that fatty acid is another differential metabolite in the poll gland during the breeding season based on non-targeted metabolomics. Our proteomic analysis revealed that the key protein CPT1A, a master regulator of fatty acid metabolism, was significantly activated, and signaling pathways related to fatty acid metabolism, including fatty acid biosynthesis, elongation, metabolism, and degradation pathways, were stimulated in the poll gland during the breeding season. Consistently, the expression pattern revealed that the central regulator CPT1A involved in fatty acid metabolism was widely distributed in the acinar cells of the poll gland, and its expression increased significantly during the breeding season, together with the results that the expression of COX IV, a well-established marker for cellular energy metabolism levels [23], ATP content, and CAT activity increased significantly during the breeding season, which may lead to vigorous fatty acid metabolism and energy generation.

Fatty acid metabolism is a central energy-producing pathway and is closely related to various physiological processes in mammals, including estrus and breeding [27,28]. Similar metabolic shifts, such as weight loss, hormone elevation, and enhanced glandular activity, have been observed in other seasonal breeders like male yaks and sheep during the breeding season [29,30]. Unlike Bactrian camels, which rely on social interactions and territorial defense, yaks and sheep engage in more direct competition, with male yaks showing significant fat and muscle breakdown due to high energy expenditure [31]. Male sheep also exhibit increased fat oxidation and elevated testosterone, fueling their aggressive behavior [32]. These shifts in fatty acid metabolism and energy supply support their reproductive efforts, while Bactrian camels show more moderate changes, reflecting different reproductive strategies. CPT1A, a mitochondrial enzyme, is a master regulator of fatty acid β-oxidation by catalyzing the transport of long-chain fatty acids into mitochondria for energy production [19]. These metabolic adjustments are key to supporting reproductive efforts across species, including courtship and mating. In Bactrian camels, exploration of the role of CPT1A-mediated fatty acid metabolism in the camel poll gland would help further understand the function of the poll gland and its important role in breeding, while also highlighting unique camel-specific features such as the importance of the poll gland in pheromone secretion [10,12]. By comparing these metabolic processes in different seasonal breeders, we can better understand which features are specific to camels and which reflect broader reproductive strategies among seasonal breeders. The elevated expression of CPT1A and COX IV and increased ATP production in the poll gland during the breeding season support the hypothesis that male Bactrian camels undergo significant metabolic reprogramming to meet the energy demands of reproduction.

Some limitations of this study should be acknowledged. While we focused primarily on fatty acid metabolism in the poll gland, exploring lipid metabolism and mitochondrial activity in other tissues, such as liver and muscle, could provide additional evidence of systemic energy mobilization during the rutting season. Investigating the expression of CPT1A and other lipid metabolism markers in these tissues would offer a broader view of metabolic shifts and their role in supporting the energy demands of reproduction.

**Figure 7:**
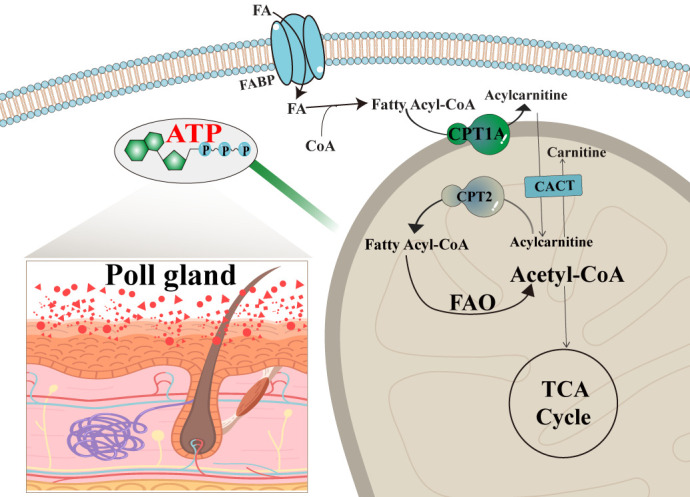
Potential molecular mechanism of CPT1A-mediated fatty acid metabolism in the poll gland of male Bactrian camels. FA: fatty acid. FAO: fatty acid oxidation. CACT: carnitine-acylcarnitine translocase. TCA: tricarboxylic acid cycle. CPT1A: carnitine palmitoyltransferase 1A. CPT2: carnitine palmitoyltransferase 2.

In summary, as shown in Figure 7, in the rutting season, male camels undergo significant metabolic changes to support reproductive physiology. Fatty acids from the circulatory system are taken up by poll gland acinar cells and activated into fatty acyl-CoA. This activated form is then converted to acylcarnitine via CPT1A in the presence of carnitine at the mitochondrial membrane. The resulting acylcarnitine is transported into the mitochondrial matrix by carnitine-acylcarnitine translocase (CACT). Inside the mitochondria, acylcarnitine is reconverted to fatty acyl-CoA, which undergoes β-oxidation to generate ATP. This energy production sustains the high metabolic demands of the poll gland during periods of heightened secretory and reproductive activity. Such findings provide a theoretical basis for understanding the reproductive patterns of camels, improving their reproductive functions, and increasing fertility rates.

## 5. Conclusions

This study identified CPT1A as a central regulator of fatty acid oxidation in the poll gland tissues of male Bactrian camels during the breeding season. Elevated CPT1A expression, along with its association with key metabolites such as acetylcarnitine and glycine, underscores its contribution to mitochondrial energy production during rut. These findings highlight the role of CPT1A-mediated fatty acid metabolism in sustaining poll gland function and reproductive success. This study advances our understanding of camelid reproductive physiology and lays the foundation for improving fertility and breeding management in this species. Future studies should focus on exploring the deeper molecular mechanisms of CPT1A and fatty acid metabolism in regulating the reproductive processes of Bactrian camels during the breeding season.

## Data Availability

The original contributions presented in this study are included in the article. Further inquiries can be directed to the corresponding author(s).

## Supplementary Materials

The following supporting information can be downloaded at https://www.mdpi.com/article/10.3390/biom15070988/s1, Table S1: The biological processes (BPs) associated with fatty acid metabolism from DIA proteomics. Table S2: The 6 BP and 119 DEP RAW data identified from DIA proteomics. Table S3: The pathways associated with fatty acid metabolism from DIA proteomics. Table S4: The 4 pathway and 22 DEP RAW data identified from DIA proteomics. Table S5: The 8 pathways related to proteomics and metabolomics. Figures S1 and S2: Whole Western blots of CPT1A and β-actin, respectively.
