# Supplementary figures and images for: Fatty Acid Metabolism via CPT1A Supports Poll Gland Function and Rutting Activities in Male Bactrian Camels
Source: Biomolecules. 2025 Jul 11;15(7):988. doi: 10.3390/biom15070988 (PMC12292770; doi:10.3390/biom15070988)

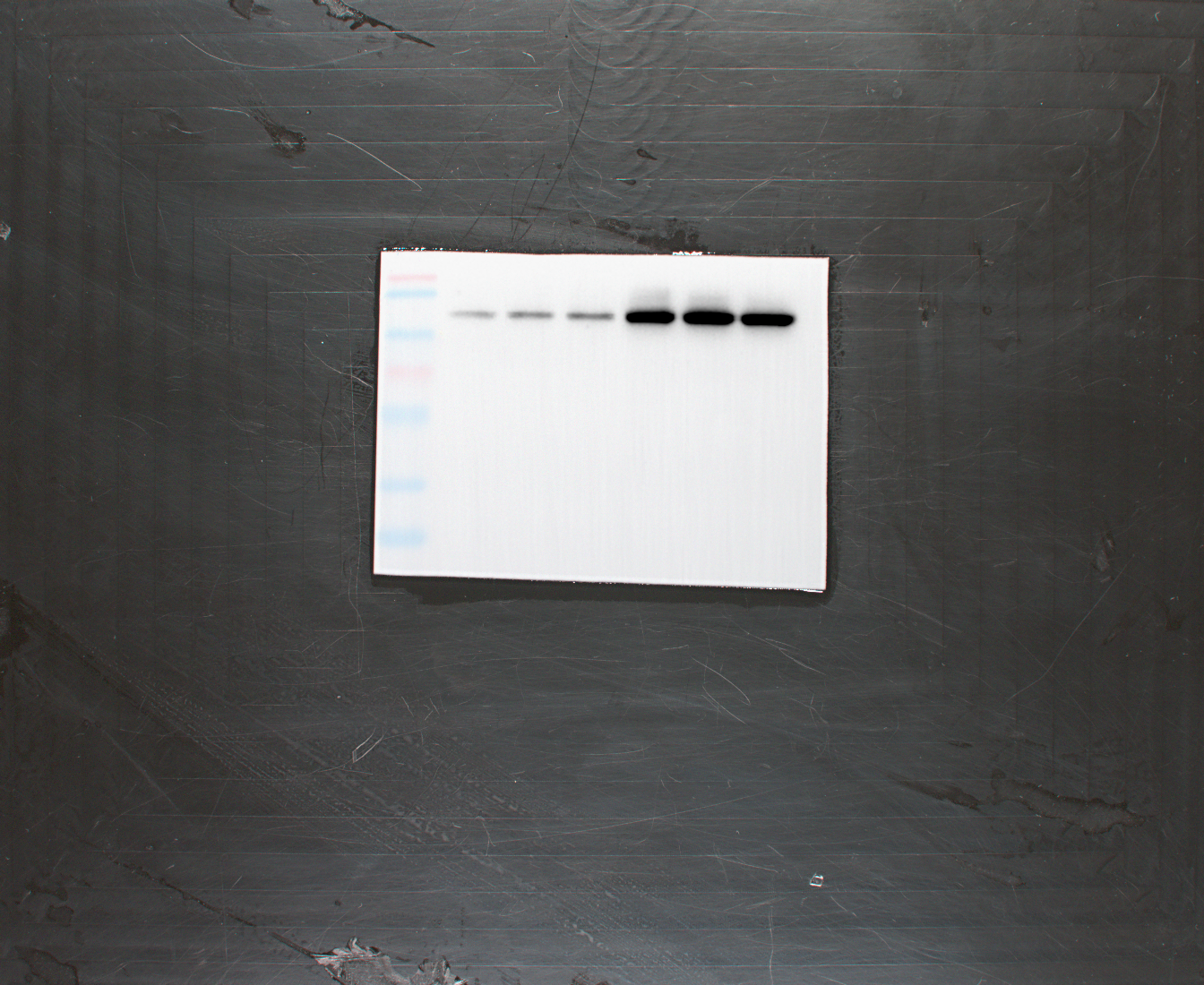

Supplement: Supplementary file 1 [file biomolecules-15-00988-s001.zip › Figure S1.Tif]

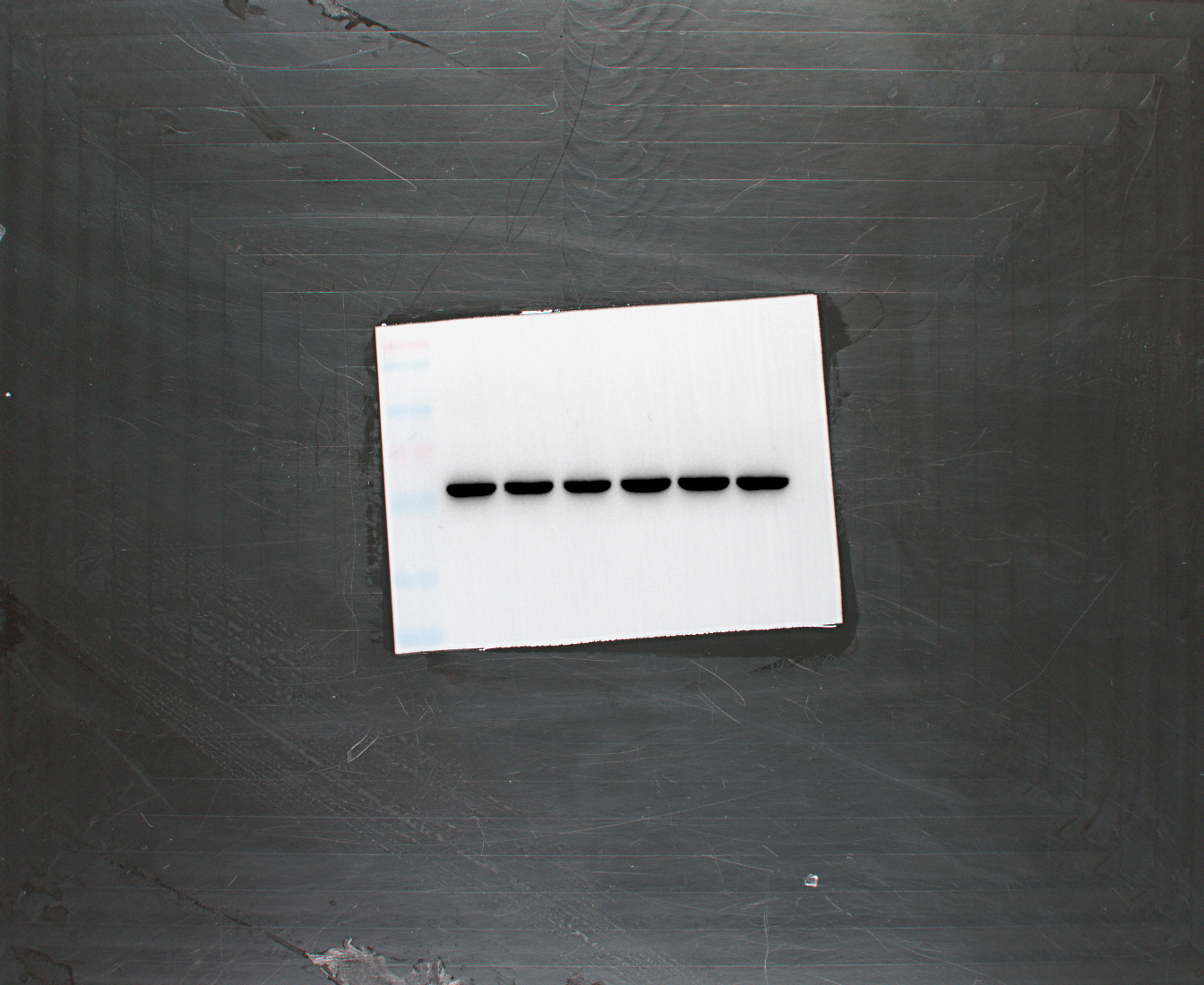

Supplement: Supplementary file 1 [file biomolecules-15-00988-s001.zip › Figure S2.Tif]
